# Successful introduction of milk after a negative double‐blind placebo‐controlled food challenge test is independent of the total dose and milk product used during the challenge test

**DOI:** 10.1002/iid3.305

**Published:** 2020-04-22

**Authors:** Celine A. van de Ven, Irene Herpertz, Lidy van Lente, Gerbrich N. van der Meulen, Arvid W. A. Kamps

**Affiliations:** ^1^ Paediatric Allergy Centre, Department of Paediatrics Martini Hospital Groningen The Netherlands; ^2^ Department of Clinical Epidemiology, Martini Academy Martini Hospital Groningen The Netherlands

**Keywords:** children, double‐blind placebo‐controlled food challenge, food allergy, oral food challenge test, success rate

## Abstract

**Background:**

Failure of milk introduction after a negative food challenge test is reported in a substantial number of patients. For this reason, guidelines recommend that the total dose of milk protein for a food challenge test should be comparable to a normal serving.

**Objective:**

Our aim is to compare the success rate of milk introduction after a negative double‐blind placebo‐controlled challenge test performed with different doses of milk protein and different milk products.

**Methods:**

We conducted a retrospective chart review of 485 patients challenged with a low or high dose of milk protein. Pasteurized milk and milk protein powder were used for the low‐dose challenge tests, and condensed milk for the high‐dose challenge tests. Successful introduction was defined as regular milk consumption, and discontinuation of further introduction due to the reappearance of symptoms as unsuccessful introduction. We also evaluated the association between milk products and successful introduction.

**Results:**

The outcome of 288 (59.4%) double‐blind placebo‐controlled food challenge tests was negative. There were no significant differences between the low and high dose of milk protein in patient characteristics, percentage of patients lost to follow‐up (15% vs 20%), in whom introduction had not yet been performed (4% vs 3.1%), reappearance of symptoms (18% vs 17%), and successful introduction (88.0% and 83.4%). Age, gender, specific immunoglobulin E for milk, dose of milk protein, and atopy were not associated with successful introduction. Children who experienced symptoms during the introduction were less likely to consume milk (*P* < .001). There was a nonsignificant trend toward higher successful introduction rate if pasteurized milk was used as test material compared to milk protein powder, and condensed milk.

**Conclusion and Clinical Relevance:**

Successful introduction of milk after a negative challenge test is independent of the total dose of milk protein, and milk product used during the challenge test.

## Graphical abstract

We evaluated the success rate of milk introduction after negative food challenge tests performed with different doses of milk protein and different milk products. Successful introduction of milk was independent of the total dose of milk protein, and milk product used during the challenge test.

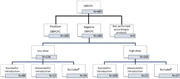

## 1 INTRODUCTION

Oral food challenge tests are performed to diagnose or exclude a food allergy, and also to evaluate whether tolerance to a specific food allergen has been acquired.^1^ If the outcome of the challenge test is negative, information is provided to the patient and parents how they can (re)introduce the specific food. The necessity to reintroduce food after a negative challenge test depends on the age of the child, and the specific food. Especially in young children, perceived reactions to milk are common, and result in unnecessary elimination of milk, and potentially dietary deficiencies.^2^

Several studies reported failure of introduction of milk in a substantial number of patients after a negative food challenge test.^3^, ^4^, ^5^, ^6^, ^7^, ^8^ Symptoms might reappear when a higher dose of the specific food is consumed at home compared to the total dose the child was exposed to during the challenge test. This might be one of the explanations for introduction failure. Indeed, international guidelines recommend that the total dose consumed during a challenge test should be comparable to a normal serving.^1^ Furthermore, it might be that the food was prepared differently compared to the product used for the challenge test. On the other hand, the reported symptoms may also be coincidental, for instance due to a viral illness.

In 2014 we increased the total dose of milk protein for double‐blind placebo‐controlled food challenge test (DBPCFC). However, after we had increased the total cumulative dose of milk protein for DBPCFC tests, caregivers still regularly reported symptoms during introduction of milk at home. This prompted us to evaluate the success rate of milk introduction after a negative challenge test with different total doses of milk protein. We were also interested in whether the use of different milk products for the challenge test affected the outcome.

## 2 METHODS

This retrospective chart review was approved by our institutional review board. All patient characteristics and outcomes of oral food challenge tests are registered in the database of our pediatric allergy center. For this study, the patient characteristics and outcomes of milk introduction at home were analyzed for all patients who had a negative DBPCFC test for milk in the period from December 2011 until October 2017. In our practice, a diagnosis of milk allergy is established by performing a DBPCFC, according to the guidelines.^1^ Specific immunoglobulin E (IgE) for milk is not routinely determined in our daily practice as the specificity and sensitivity are fairly low.^1^, ^9^

### 2.1 Double‐blind placebo‐controlled food challenge

DBPCFC tests were conducted according to the European Academy of Allergy and Clinical Immunology (EAACI) guidelines.^1^ All foods for the DBPCFC were prepared by the institution's nutrition staff. From December 2011 until April 2014 a total dose of 2.2 g of milk protein was used. From April 2014 a total dose of 4.4 g of milk protein was used for the food challenge tests. The cumulative dose of 2.2 and 4.4 g will be referred to as low and high dose, respectively. The total amount of milk protein was administered in the course of 4 hours, with 30 minutes between each fractional dose. Unblinding of the test was performed 2 days after the second test day.

For the low‐dose DBPCFC tests, spray dried milk protein powder (Protifar, Nutricia, The Netherlands), and pasteurized milk were used. The recipes for these tests have been validated. To the best of our knowledge, validated recipes for high‐dose DBPCFC tests are not available. We chose to use condensed milk (Balance coffee creamer; Friesche Vlag, The Netherlands) for the high‐dose DBPCFC tests. Condensed milk contains a substantially higher amount of milk protein (10.5 g/100 g) compared to whole milk (3.7 g/100 g). This was the rationale to use condensed milk as it enabled us to obtain a normal serving for young children.^10^

### 2.2 Criteria for the outcome of the oral food challenge test and severity of symptoms

Observed symptoms were classified according to international guidelines.^1^ According to protocol, the challenge test was considered positive if objective symptoms occurred. If mild symptoms, such as oral discomfort or abdominal pain occurred and did not worsen in the next 30 minutes, patients were encouraged to eat or drink the same dose again. If the same symptoms occurred, the test was considered positive. Otherwise, the stepwise increments were continued. All DBPCFC tests were conducted by specially trained nurses.

After a negative DBPCFC test, parents were provided a comprehensive introduction scheme. A follow‐up visit to the clinic or by telephone consultation was planned within 6 months after the challenge test. Caregivers of the children who appeared to be tolerant for unheated milk were asked whether or not milk was regularly consumed.

### 2.3 Primary and secondary outcome

Primary outcome was the percentage of successful introduction of milk after a negative DBPCFC test in both dose groups. All patients who regularly consumed milk products as advised after a negative food challenge test, were allocated to the “successful introduction” group. Unsuccessful introduction was defined as patients whose caregivers discontinued introduction of milk at home. The secondary outcome was the association between milk product and successful introduction.

### 2.4 Statistical analysis

Nominal data are presented as frequencies (percentages). Ordinal variables are presented as median (interquartile range), and continuous variables as mean (standard deviation, SD) or median (interquartile range). Statistical significance was analyzed for categorical variables using Fisher's exact test. Based on clinical experience, a predefined set of potentially relevant predictors of the outcome successful introduction were selected and examined by logistic regression analysis. *P*< .05 were considered statistically significant. All analyses were performed using SPSS software (SPSS Inc, Chicago, version 20).

## 3 RESULTS

### 3.1 Patient characteristic

**Figure 1:**
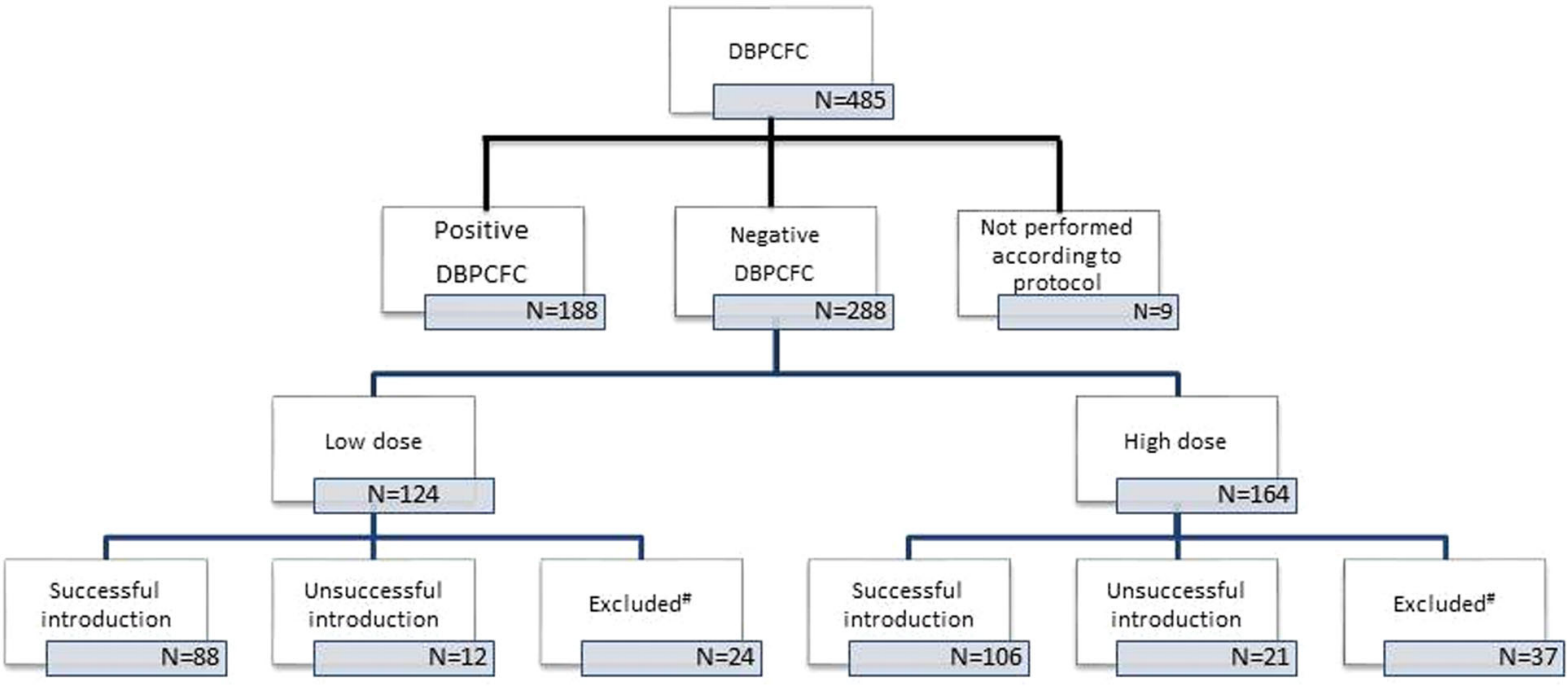
Outcome for introduction at home after a negative DBPCFC in patients challenged with low‐ and high‐dose milk. ^#^Excluded = lost to follow‐up, milk not yet introduced, and introduction scheme not completed. DBPCFC, double‐blind placebo‐controlled food challenge

The result of the DBPCFC test was negative in 288 (59.4%) of 485 analyzed tests. Of the 288 tests, 124 (43.1%) and 164 (56.9%) were performed with the low‐ and high‐dose milk protein, respectively (Figure 1). In one patient in the low‐dose group, and three patients in the high‐dose group milk was partially introduced at home or only baked milk was introduced. These patients were excluded from further analysis. Characteristics of the patients were comparable in both groups (Table 1).

**Table 1:** Patient characteristics of the children with a negative outcome of DBPCFC

	Low dose	High dose
	*N* = 124	*N* = 164
Age^a^	12 (10)	11 (11)
0‐2 y, %	99 (80%)	134 (82%)
2‐4 y, %	18 (14%)	19 (11%)
Over 4 years of age, %	7 (6%)	11 (7%)
Sex (% of boys)	64 (52%)	108 (66%)
Multiple food allergies, %^b^	10 (8%)	8 (5%)
History of eczema or wheezing, %	50 (40%)	79 (48%)
Family member with eczema, allergy, or asthma, %^c^	84 (68%)	124 (76%)
Specific IgE milk >0.35 kU/L, %^d^	30 (24%)	26 (16%)

Abbreviations: DBPCFC, double‐blind placebo‐controlled food challenge; IgE, immunoglobulin E; IQR, interquartile range.

^a^ Age presented as median in months (IQR).

^b^ Multiple allergies: allergy to another food allergen than milk determined by a challenge test.

^c^ Family member (first degree) with eczema, allergy, or asthma.

^d^ Information was available for 43 (35%) and 40 (24%) in the low‐ and high‐dose group, respectively.

### 3.2 Symptoms observed during DBPCFC tests with negative outcome

During the challenge tests, symptoms were observed in a substantial number of patients in the low‐ (12%) as well as the high‐ (25%) dose group on the placebo as well as the verum day. These symptoms were all mild and transient (erythema, discomfort, and sneezing), and did not reoccur after continuation of the challenge test. These tests were all completed, and classified as negative.

### 3.3 Symptoms reported during introduction of milk

After the low‐dose DBPCFC test, 19 (15%) patients did not return for follow‐up or scheduled telephone consultation. In four children milk had not yet been introduced at the time of follow‐up (Figure 1). Thus, we analyzed the outcome of 100 patients in the low‐dose group. Successful introduction was achieved in 88 (88%) of the patients. Out of these 88, 16 (18.2%) caregivers reported symptoms. In 12 (12%) of the patients, the caregivers discontinued the introduction of milk due to erythema, discomfort, or behavioral changes. In two patients antihistamines were administered by the caregivers.

In the high‐dose group, 32 (20%) were lost to follow‐up. In two children, milk was not introduced at the time of follow‐up (Figure 1). The outcome of 127 patients in the high‐dose group was analyzed. A successful introduction was achieved in 106 (83.5%), although 18 (17.0%) of these caregivers reported the occurrence of symptoms during home introduction of milk. In 21 (16.5%) patients, the caregivers decided to discontinue further introduction of milk. The most reported symptoms were erythema, urticaria, and discomfort. In 5 of the patients antihistamines were administered by the caregivers.

In both groups, none of the caregivers contacted the general practitioner or allergy center or at the time the symptoms occurred. They reported the symptoms during the follow‐up visit. The percentage of successful introduction was not significantly different between both groups.

### 3.4 Association of milk product and successful introduction

**Figure 2:**
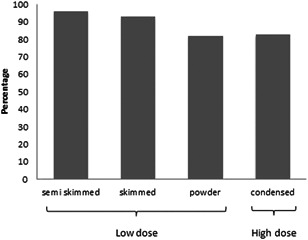
Percentage of successful introduction of milk at home per milk product used during the challenge test. Powder = milk protein powder (Protifar)

For the low‐dose DBPCFC test skimmed milk, semi‐skimmed milk and milk protein powder (Protifar, Nutricia, The Netherlands) were used as milk protein. For the high‐dose DBPCFC test only condensed milk was used. Although there was a trend toward higher introduction failure with milk protein powder, and condensed milk, this was not statistically significant (Figure 2).

### 3.5 Association of patient characteristics and successful introduction

The following patient characteristics were identified as potential predictors: age, gender, specific IgE for milk, atopy, milk product, dose, and symptoms during the DBPFC or introduction. These characteristics were not associated with the outcome of the introduction of milk at home, except for the amount of symptoms during the introduction and the outcome of the introduction indicating that children who develop symptoms during the introduction at home were less likely to consume milk products after a negative food challenge test compared to children who did not report any symptoms during the introduction at home (*P* < .001) (Table 2).

**Table 2:** Potential predicting factors for the outcome of the introduction of milk at home after a negative DBPCFC

	*P*‐value*	OR	95% CI
Gender	.94	1.03	0.49–2.17
Milk product	.33		
*Reference* (*condensed milk*)			
Semi‐skimmed	.34	2.77	0.35–22.25
Protifar	.98	0.99	0.42–2.33
Skimmed	.11	5.35	0.69–41.56
Age in months	.91		
*Reference* (*0–12*)			
12‐24	.60	1.26	0.53–3.00
24‐60	.83	0.89	0.30–2.63
60+	.78	0.80	0.16–4.01
Challenge dose	.34	1.45	0.68–3.12
Symptoms during DBPCFC	.57	0.93	0.71–1.21
Symptoms during introduction	<.001	0.27	0.18–0.40
History of eczema and/or wheezing	.56	1.26	0.58–2.75
Family members with eczema, asthma or allergy	.38	2.40	0.54–10.72
IgE milk	1.0	0.94	0.30–2.94

Abbreviations: DBPCFC, double‐blind placebo‐controlled food challenge; IgE, immunoglobulin E; OR, odds ratio; 95% CI, 95% confidence interval.

* *P*‐values based on univariate analyses by *χ* ^2^ or logistic regression. Odds ratio based on successful introduction.

## 4 DISCUSSION

In this retrospective study we demonstrated that successful introduction of milk did not significantly change after we started to perform challenge tests with a higher cumulative dose of milk. Successful introduction of milk was achieved in the majority of children after a negative challenge test with low (88%) or high (83%) doses of milk protein. However, up to 30% of the caregivers reported symptoms during introduction of milk at home. Although only mild symptoms were reported, approximately 15% of the caregivers discontinued further introduction of milk. Successful introduction at home could not be predicted by patient characteristics or observed symptoms during the challenge test. Although not statistically significant, introduction failure occurred more frequently if children had been challenged with milk protein powder or condensed milk.

Successful introduction after a negative challenge test for milk has been reported to vary from 68% to 90%.^3^, ^4^, ^5^, ^6^, ^7^ In our study successful introduction was achieved in 88% of patients in the low‐dose group and in 83.4% of patients in the high‐dose group. Differences in successful introduction might be due to a different cumulative dose of milk protein or different milk product used to perform the challenge test. Only Dambacher specified the milk product used for the challenge test.^6^ The percentage of successful introduction after a challenge test performed with 4.4 g milk protein powder in Dambacher's study (81%) was comparable to the success rate in our study for patients challenged with a cumulative dose of 2.2 g milk protein powder (82%). This suggests that the dose of milk protein used during the challenge test does not affect introduction of milk after a negative challenge test. Another explanation might be that the dose of milk introduced at home is higher than the cumulative dose during the DBPCFC test. van der Valk et al^4^ used a total cumulative dose of 0.57 milk protein, which might explain the lower percentage of successful introduction. In addition, it might be that for some children, the threshold to respond, is higher than the cumulative dose of milk protein used in our challenge test.

In our study, 12% and 16% of the parents reported symptoms for patients challenged with a low and high dose, respectively. Reappearance of symptoms during introduction at home was reported in 12% to 43% of the patients in earlier studies.^4^, ^6^, ^7^ van der Valk et al^4^ reported that successful introduction was significantly lower if symptoms had occurred during the challenge test. In our study, successful introduction was not associated with the occurrence of symptoms during the challenge test. Fear of occurrence of symptoms during introduction might be predictive for unsuccessful introduction. Due to the retrospective design of our study, we could not determine this issue as we implemented quality‐of‐life questionnaires to our standard care only recently.^11^

A limitation of our study is the fairly high number of patients lost to follow‐up (approximately 20%). It remains unknown whether these children were exposed to milk products or not. Interestingly, Schrijvers et al recently reported that successful introduction of milk after a negative food challenge test is not affected by the way follow‐up was conducted (no follow‐up, follow‐up in person or by telephone).^3^

We need to clarify why we did not determine specific IgE in the majority of patients. The guidelines state that other diagnostic procedures such as skin prick testing, or measurement of specific IgE may have to be considered as food challenge tests are time consuming and not without risk to the patient.^1^ However, the specificity and sensitivity of skin prick tests and specific IgE are fairly low.^9^ Furthermore, it has been demonstrated that a substantial number of children have non‐IgE‐mediated milk allergy.^12^ To this end, in our allergy clinic with sufficient resources to perform DBPCFC, we do not routinely determine specific IgE for milk.

Another limitation might be that the effect of different milk processing procedures on the allergenicity is not completely unraveled.^13^ It is likely that the allergenicity of the provoking material we used for the challenge tests are not comparable and might differ from the allergenicity of the milk products used at home. It has been shown that whey proteins denature by heat treatment unlike casein proteins.^14^ As casein proteins account for up to 80% of the total proteins in milk, it has been hypothesized that heating of milk does not substantially reduce allergenicity.^15^ Indeed, pasteurization has been shown not to affect the allergenicity of milk. The used skimmed milk and semi‐skimmed milk are pasteurized milk products. Sterilization only partly reduces allergenicity.^16^ Condensed milk is obtained by sterilization and evaporation of milk. Milk protein powder (Protifar) is obtained by spray drying of milk. It is unknown how evaporation or spray drying of milk affects allergenicity.^13^ Due to intensive processing, condensed milk may well be less allergenic than pasteurized or sterilized milk products used at home. This might explain the higher percentage of introduction failure after a negative challenge test in which condensed milk was used as a provoking material.

## 5 CONCLUSION

Successful introduction of milk after a negative DBPCFC test was not increased if a higher cumulative dose of milk protein was used during the challenge test. Up to 30% of caregivers reported symptoms during home introduction, and approximately 15% discontinued further introduction. These results were independent of the total dose and milk products used during the DBPCFC. These findings suggest that we may need to perform challenge tests with a higher total dose of milk protein, and a milk product comparable to the milk product used in daily life of the child.

## CONFLICT OF INTERESTS

The authors declare that there are no conflict of interests.

## AUTHOR CONTRIBUTIONS

Celine van de Ven had primary responsibility for protocol development, data analyses, and writing the manuscript. Irene Herpertz had primary responsibility for protocol development, and contributed to the writing of the manuscript. Lidy van Lente participated in the development of the protocol and analytical framework for the study, supervised the design, and execution of the study, and contributed to the writing of the manuscript. Gerbrich van der Meulen participated in the development of the protocol, and contributed to the writing of the manuscript. Arvid Kamps had primary responsibility for protocol development, outcome assessment, and writing the manuscript.

## ETHICS STATEMENT

This retrospective chart review was approved by the Medical Ethics Committee of the Martini Hospital Groningen, The Netherlands (MEC 2016‐046).

## Data Availability

The data that support the findings of this study are available from the corresponding author upon reasonable request.
